# Development of real-time reverse transcription recombinase polymerase amplification (RPA) for rapid detection of peste des petits ruminants virus in clinical samples and its comparison with real-time PCR test

**DOI:** 10.1038/s41598-018-35636-5

**Published:** 2018-12-10

**Authors:** Yuanli Li, Lin Li, Xiaoxu Fan, Yanli Zou, Yongqiang Zhang, Qinghua Wang, Chengyou Sun, Shude Pan, Xiaodong Wu, Zhiliang Wang

**Affiliations:** 1 OIE Reference Laboratory for Peste des Petits Ruminants, National Research Center for Exotic Animal Diseases, China Animal Health and Epidemiology Center, Qingdao, Shandong P. R. China; 2 College of Animal Husbandry and Veterinary Medicine, Shenyang Agricultural University, Shenyang, Liaoning P. R. China

## Abstract

Peste des petits ruminants (PPR), caused by small ruminant morbillivirus (SRMV), formerly called peste des petits ruminants virus (PPRV), is one of the most important pathogens in small ruminants, and has tremendous negative economic impact on the sheep industry worldwide. Current detection of PPRV in clinical samples mainly relies on real-time RT-PCR. Particularly, samples collected from rural area require highly equipped laboratories for screening. A rapid, real-time reverse-transcription recombinase polymerase amplification assay (RT-RPA), employing primers and exo probe, was thus developed to perform at 42 °C for 20 min, and the detection limit at 95% probability was 14.98 copies per reaction and 0.326 TCID_50_/mL based on plasmid copy number and tissue culture infectivity titre. All the four lineages of PPRV could be detected with no cross-reaction to other pathogens including measles virus (MeV), goatpox virus (GTPV), canine distemper virus (CDV), foot-and-mouth disease virus (FMDV) and *Mycoplasma capricolum* subsp*. capripneumoniae* (*Mccp*). The performance of real-time RT-RPA assay was validated by testing 138 field samples and compared to real-time RT-PCR. The results indicated an excellent diagnostic agreement between real-time RT-RPA and a reference real-time RT-PCR method with the kappa value of 0.968. Compared to real-time RT-PCR, the sensitivity of real-time RT-RPA was 100%, while the specificity was 97.80%. The developed RT-RPA assay offers a promising platform for simple, rapid, and reliable detection of PPRV, especially in the resource-limited settings.

## Introduction

Peste des petits ruminants (PPR), caused by small ruminant morbillivirus (SRMV), formerly called peste des petits ruminants virus (PPRV), mainly affects sheep, goats and wild small ruminants, leading to high mortality^1^. This acute, highly contagious viral disease has severely impacted the farmer’s livelihood and led to a constantly feared threat to socioeconomic development of endemic regions, including Africa, the Middle East, Arabian Peninsula and Southern Asia, since its emergence in West Africa in 1942. PPR is categorized as a notifiable disease by World Organisation for Animal Health (OIE) and emerges as the priority of global strategy for the control and eradication^2^.

PPRV, a negative-sense, single-stranded RNA virus, belongs to genus *Morbillivirus*, family *Paramyxoviridae*, the same group of which also contains measles virus, rinderpest virus, canine distemper virus, phocine distemper virus, dolphinmorbillivirus and felinemorbillivirus. The genome of PPRV, 3′-N-P-M-F-H-L-5′, in turn encodes structural proteins nucleocapsid, phosphoprotein, matrix, fusion, haemagglutinin, large polymerase. The PPRV strains have been classified into four different lineages (I, II, III, and IV) based on the partial sequences of the N and the F genes^3^. Conventional reverse transcription polymerase chain reaction (RT-PCR) and real-time RT-PCR were successfully developed and widely accepted as standard diagnostic tests for PPRV^4^. Additionally, recent evidence showed that loop mediated isothermal amplification (LAMP) presented an isothermal technology with favorable convenience, sensitivity and specificity^5^. However, limitations still exist that RT-PCR or real-time RT-PCR is comparatively time-consuming and requires expensive equipment while the complexity of primers design in LAMP affects its further comprehensive use.

Recently, recombinase polymerase amplification (RPA) has emerged as a rapid detection on nucleic acid for the diagnosis of diverse pathogenic microorganisms, such as HIV^6^, Zika virus^7^, listeria monocytogenes^8^, giardia^9^, cryptosporidium^10^ and Entamoeba^11^. As an isothermal alternative to PCR or real-time PCR, RPA incorporates three core enzymes, a recombinase, a single-stranded DNA-binding protein (SSB) and strand-displacing polymerase, which orchestrate DNA synthesis from primer-paired target DNA. The products of RPA can be detected by gel electrophoresis, real-time monitored with exo or fpg probes, or visualized with lateral flow dipstick (LFD) and nfo probes^12,13^. Of note, RPA reaction requires the optimal temperatures of 37–42 °C, 10–20 min, and performs similar or even higher sensitivity compared to PCR or LAMP, making it as a potential candidate for portable diagnostic approach in the field. In this study, we aim to develop real-time reverse transcription recombinase polymerase amplification (RT-RPA) for the rapid detection of PPRV from clinical samples and evaluate its efficacy in comparison with real-time PCR testing.

## Results

### Optimization of PPRV real-time RT-RPA

**Figure 1:**
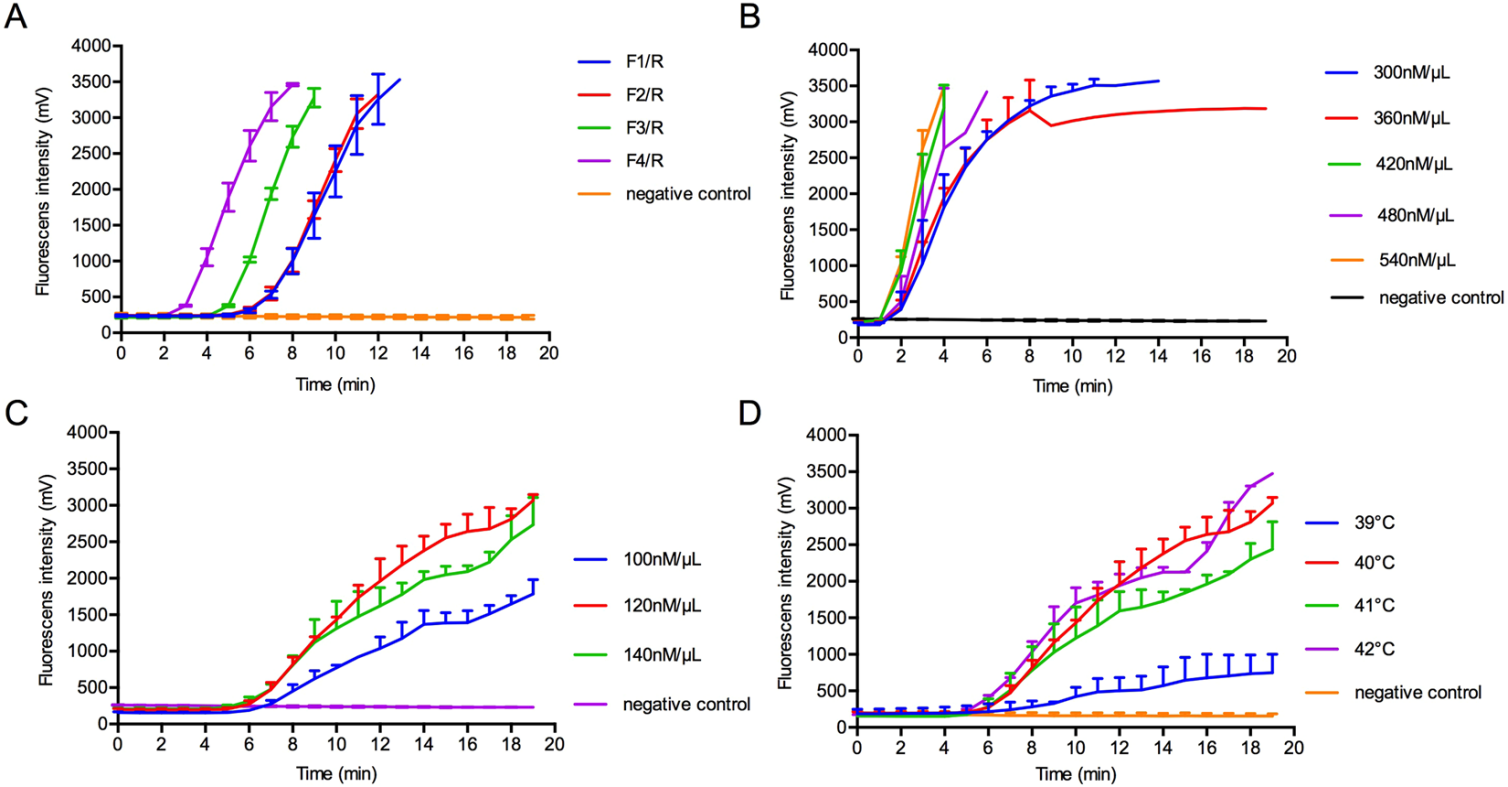
Optimum condition of real-time RT-RPA. Amplification efficacy was determined by using different groups of primers and probe (**A**); primers at concentration of 300, 360, 420, 480, 540 nmol/μL (**B**); probe at concentration of 100, 120, 140 nmol/μL (**C**); at temperatures of 39 °C, 40 °C, 41 °C, 42 °C (**D**).

In order to optimize the real-time RT-RPA reaction, 4 assemblies of primers and probe were designed and the fluorescence signals were detected. The amplification efficiency of group F4/R was the highest among all candidates during testing RNA of 2 × 10^3^ TCID_50_/mL PPRV (Fig. 1A). Based on the fluorescence intensity and its corresponding time, 420 nmol/µL forward and reverse primers, along with 120 nmol/µL probe were ultimately deployed in our assay with the operating temperature of 42 °C (Fig. 1B–D).

### Sensitivity and specificity of PPRV real-time RT-RPA

**Figure 2:**
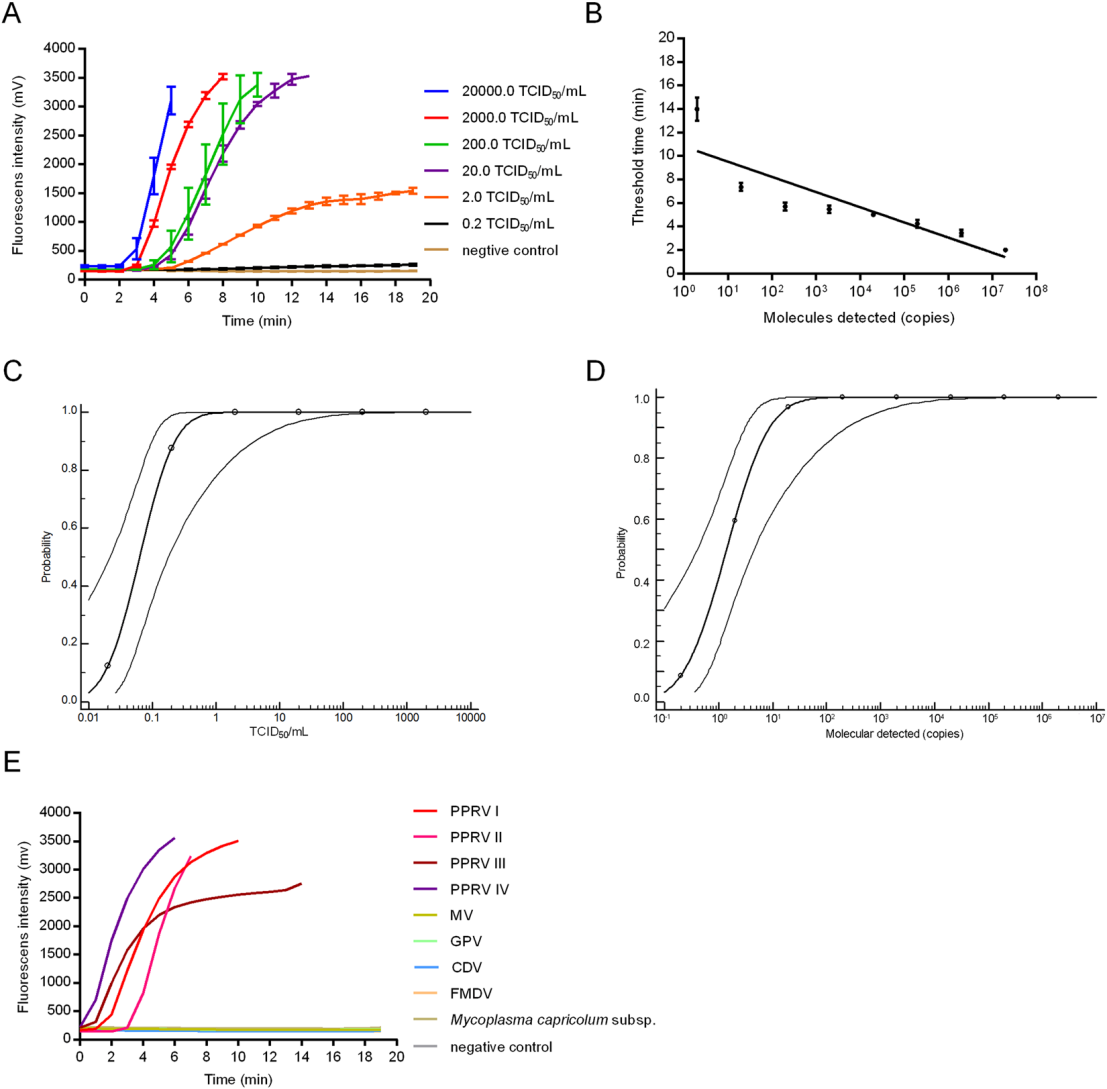
The sensitivity and specificity of real-time RT-RPA. (**A**) Typical raw fluorescence data of real-time RT-RPA assay for series dilution of PPRV (TCID_50_/mL). (**B**) PPRV RNA molecules after series dilution were detected by real-time RT-RPA assay (copies per reaction). Probit regression analysis using MedCalc Software was performed on data of 8 replicates from serial dilutions of an infected cell culture (**C**) and PPRV RNA genome (**D**). (**E**) Specificity test result of real-time RT-RPA on detecting four lineages of PPRV, MeV, GTPV, CDV, FMDV and *Mccp*.

To determine the sensitivity of PPRV real-time RT-RPA, a dilution ranging from 2 × 10^4^ to 0.2 TCID_50_/mL was tested for eight replicates. As shown in Fig. 2A, remarkable increase of fluorescence intensity was observed by the detection from 2 × 10^4^ to 2 TCID_50_/mL at 42 °C within 20 min. A 10-fold serial dilution of the *in vitro* transcribed RNA molecules from 2 × 10^6^ to 0.2 copies per reaction was simultaneously detected with the same method (Fig. 2B). Of note, our data on probit regression analysis indicated that the detection limit of PPRV real-time RT-RPA at 95% probability was 0.326 TCID_50_/mL (0.130~11.338 TCID_50_/mL, 95% CI) and 14.99 copies per reaction (4.62~902.77 copies per reaction, 95% CI), respectively (Fig. 2C,D). In order to identify the specificity of RT-RPA assay, PPRV, MeV, GTPV, CDV, FMDV and *Mccp* were involved in the specificity test. We found positive results by the detection of PPRV from lineage I, II, III and IV, while no cross reaction of the other microbes was shown (Fig. 2E).

### Performance of PPRV real-time RT-RPA assay on clinical samples and its comparison with real-time RT-PCR testing

**Figure 3:**
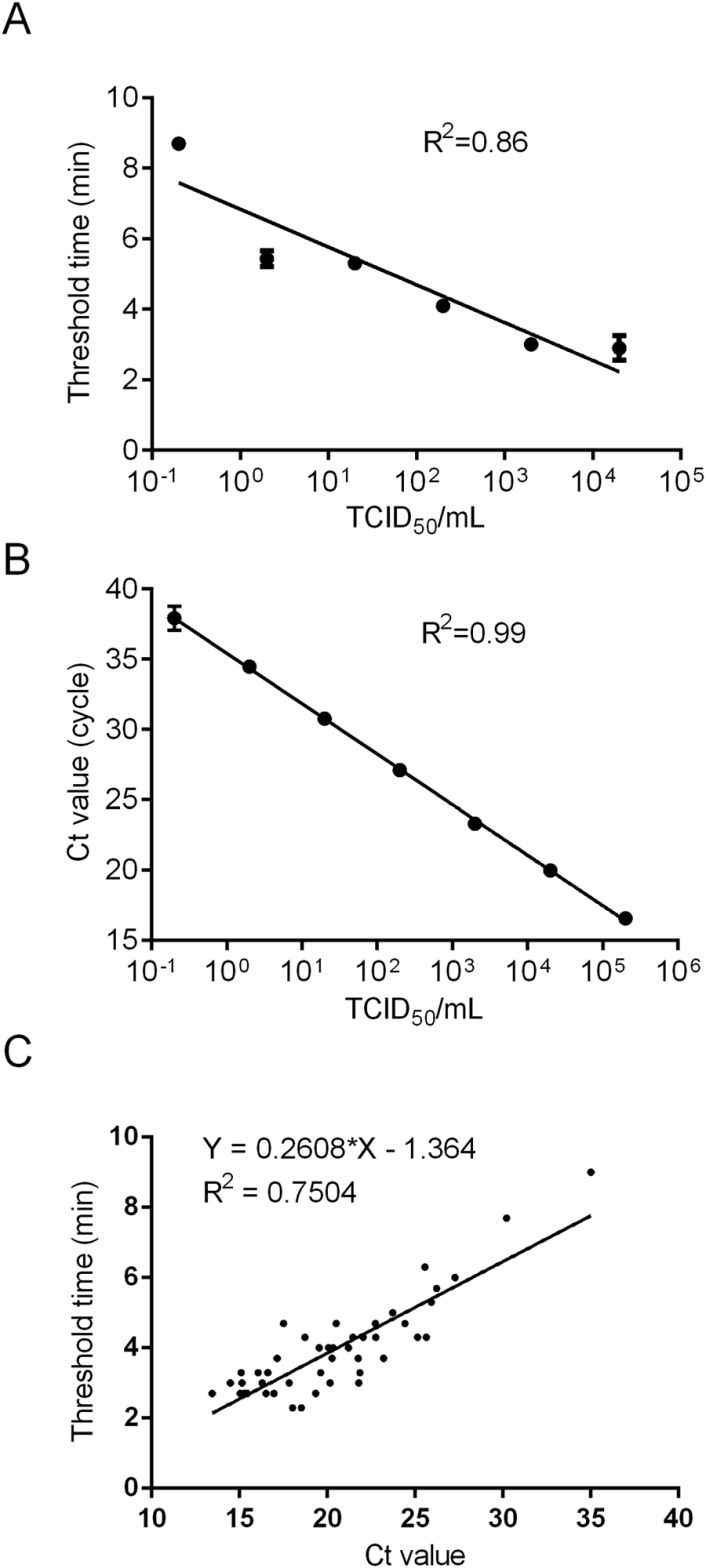
Performance of real-time RT-RPA in comparison of reference real-time RT-PCR. Reproducibility of PPRV real-time RT-RPA assay (**A**) and real-time RT-PCR (**B**) was conducted by using Prism Graphpad 5.0 software. (**C**) Comparison of clinical performance between the threshold time of PPRV real-time RT-RPA (y axis) and Ct value of real-time RT-PCR (x axis) on positive field samples (n = 47).

In order to evaluate the practical application of PPRV real-time RT-RPA, the correlation analysis was deployed to assess the performance of real-time RT-RPA and real-time RT-PCR on a dilution from 20000 to 0.2 TCID_50_/mL. Higher correlation level between cycle threshold (Ct) value and virus titer (R^2^ = 0.99) in real-time RT-PCR was found, compared to that between threshold time and virus titer (R^2^ = 0.86) in RT-RPA (Fig. 3A,B). We then determined the efficacy of PPRV real-time RT-RPA assay on the detection of clinical samples, which was also compared with real-time RT-PCR testing. The result showed that, among 138 clinical samples from sheep and goats suspected for PPR, 49 samples were identified as positive by real-time RT-RPA (threshold time, ranging from 2.3 min to 17.7 min), whereas 47 were then validated as positive by real-time RT-PCR as well (Ct value, ranging from 13.44 to 27.27) (Tables 2 and 3). PPRV RNA positive rate was 35.51% (49/138) by RT-RPA and 34.06% (47/138) by real-time RT-PCR. The results indicated an excellent diagnostic agreement between real-time RT-RPA and a reference real-time RT-PCR method with the kappa value of 0.968 (0.924~1, 95% CI). Linear correlation between Ct value of real-time RT-PCR and threshold time of RT-RPA was presented in Fig. 3C (R^2^ = 0.79). Furthermore, based on the detection result of clinical samples, the sensitivity and the specificity of RT-RPA assay for identification of PPRV were 100% (92.45~100%, 95% CI) and 97.80% (92.29~99.73%, 95% CI), respectively, in comparison to real-time RT-PCR (Tables 2 and 3).

## Discussion

PPR represents a notifiable disease in the OIE Terrestrial Animal Health Code, and countries are obligated to report the disease to the OIE according to the criteria. PPRV antigens can be detected by immunocapture ELISA (ICE), counter immunoelectrophoresis (CIEP) or agar gel immunodiffusion (AGID)^14^. Immunofluorescence and immunochemistry are also used on conjunctival smears and tissue samples collected at necropsy. Particularly, nucleic acid amplification that mainly involves conventional RT-PCR and quantitative real-time RT-PCR significantly improves molecular methods for the detection of PPRV RNA in nasal and mouth discharges, tissues and anticoagulant-treated blood^15,16^. This study describes the development of real-time RPA, a new isothermal nucleic acid amplification targeting PPRV genome. Of note, the whole process of RT-RPA merely requires 20 min at 42 °C, which dramatically shortens the reaction time of real-time RT-PCR (~100 min) and simplifies the thermal cycles. Previous studies showed that RT-RPA assays on the detection of porcine epidemic diarrhea virus (PEDV)^17^, porcine reproductive and respiratory syndrome virus (PRRSV) were performed at 40 °C for 20 min^18^, while within the same method, the rapid diagnosis of Canine distemper virus (CDV) was developed at 40 °C for 3–12 min^19^, which were the less-time consuming and consistent with our test.

In addition, the copies of target gene have been often implicated in the evaluation of sensitivity in RPA. For instance, the detection limit of real-time RPA on Capripox virus (CaPV) was 300 copies per reaction within 20 min at 38 °C^20^. The analytical sensitivity of RT-RPA was 31.8 copies *in vitro* transcribed CDV RNA and 23 copies per reaction *in vitro* transcribed virus RNA^19^. Particularly, for the detection of PPRV, around 100 copies per reaction with 95% reliability were shown in the sensitivity of the developed real-time RT-RPA^21,22^. However, most of the RPA assays failed to determine the virus quantification by TCID_50_, EID_50_ or LD_50_. Notably, in addition to the determine the lowest analyte concentration of the assay (genome copies), our study with real-time RPA method for the first time assessed the minimum detection of virus titer by using TCID_50_ and the result showed that the detection limit at 95% probability was 0.326 TCID_50_/mL. As previous evidence on the diagnosis of PPRV revealed that the detection limits of hydrolysis probe (TaqMan) real-time RT-PCR and conventional RT-PCR were approximately 0.1 and 1 TCID_50_, respectively, according to a serial dilution of the live-attenuated PPR vaccine virus^23^, the detection limits in our study were similar to real-time RT-PCR or RT-PCR.

The clinical performance of real-time RT-RPA was determined by the detection of a cohort of 138 clinical samples while real-time RT-PCR testing was conducted in parallel. Our data showed an excellent diagnostic agreement between real-time RT-RPA and a reference real-time RT-PCR method with the kappa value of 0.968, which was similar to the comparison result of RT-RPA DENV^24^. Our data is also consistent with previous data that real-time RT-RPA assay of PPRV was comparable to that of the real-time RT-PCR assay (99.4%, 161/162)^21^. Linear regression analysis on positive samples between threshold time of RT-RPA and Ct value of real-time RT-PCR was further presented and it showed close relationship between the values of two indicators (R^2^ = 0.79), suggesting that higher titer of virus corresponds to shorter threshold time and lower Ct value. However, we note that the analysis of field samples gave rise to 2 results positive with RT-RPA and negative with real-time RT-PCR in our study was also found between RT-RPA and RT-qPCR. The sensitivity of laboratory tests may vary due to the methods themselves. For instance, qPCR is generally more sensitive than PCR assay^25^. The difference of sensitivity is probably implicated between real-time RT-RPA and real-time RT-PCR. We cannot exclude these two results were false positive, for neither sequencing nor virus isolation was available for confirmatory validation from the samples with relatively small amount of virus. Preferably, diagnostic agreement between RT-RPA and RT-qPCR was analyzed by semi-log regression analysis and probit regression analysis and we found the sensitivity and the specificity of RT-RPA assay for identification of PPRV were 100% (92.45~100%, 95% CI) and 97.80% (92.29~99.73%, 95% CI), respectively, compared to the reference real-time RT-PCR method. Besides, cutoff based on the detection of a large number of samples is worthy of further consideration regarding the application of RPA in clinical use. Taken together, the real time RT-RPA assay presented a promising tool for PPRV detection, which is simple, rapid and reliable in resource-limited diagnostic laboratories and on-site facilities.

## Methods

### Virus and Mycoplasma strains

PPRV strains China/XJ/2013 (lineage IV)^26^ and Nigeria 75/1 vaccine strain (lineage II) were propagated in Vero-SLAM cells. Other viruses used in this study including measles virus (MeV), goatpox virus (GTPV), *Mycoplasma capricolum* subsp*. capripneumoniae* (*Mccp*), canine distemper virus (CDV), and foot and mouth disease virus (FMDV) were preserved in National Research Center for Exotic Animal Disease, China Animal Health and Epidemiology Center.

### Nucleic acid extraction

PPRV was titrated on Vero cells in a 96-well micro titre plate using standard cell culture procedure and the virus titre (TCID_50_/ml) was calculated using Reed and Muench formula^27^, by which, Vero cells were plated with addition of serial dilutions of the virus and the percentage of infected cells was then manually observed and recorded for each virus dilution. The total viral RNA or genomic DNA of *Mccp* were extracted from 200 µL of samples (supernatant of infected cell culture, clinical nasal/oral swabs, or tissue homogenates diluted 1:10 in phosphate-buffered saline (PBS, pH 7.4)) by using Magnetic beads pre-filled viral nucleic acid extraction kit (Tian Long Science and Technology, Xi’an, Shanxi, China) according to the manufacturer’s instructions. The total nucleic acid was eluted using RNase-free water in a final volume of 100 µL and stored at −80 °C until further use for all assays in this study.

### Design of PPRV-specific primers and probe for RPA

According to the lineages by N gene, the 39 genome sequences of PPRV lineage I (accession number: EU267273.1, KP789375.1), lineage II (accession number: HQ197753.1, X74443.2, KR781450.1, EU267274.1, KR828814.1, KR781449.1, KJ466104.1, KM212177.1, KU236379.1, KR781451.1), lineage III (accession number: KM463083.1, KJ867543.1, KJ867540.1, KJ867545.1, KJ867544.1) and lineage IV (accession number: KP868655.1, KM089832.1, KM089830.1, KX354359.1, KM091959.1, KM089831.1, KM816619.1, KP260624.1, KT633939.1, FJ905304.1, JX217850.1, KR261605.1, KX033350.1, KT270355.1, KR140086.1, KJ867542.1, KF727981.2, NC006383.2, AJ849636.2, KC594074.1, KJ867541.1, KR828813.1) strains was retrieved from the GenBank and aligned using the software MEGA 7. The PPRV-specific RPA primers and exo probe used for RT-RPA were designed from the highly conserved consensuses of the whole genomes of all four lineages according to RPA guidelines (TwistDx, Cambridge, UK). The fragment of P gene (PPRV FJ905304.1, 2112–2267) was amplified. All the primer and exo probe were synthesized and provided by Sangon (Sangon Biotech, Shanghai, China) (Table 1).

**Table 1:** RPA primers and probe.

Name^a^	Sequence (5′ to 3′)^b^	Genome location (FJ905304.1)
PPRV-RPA F1	ACTCTCAAGTACAGCGTTACYATGTTTATAG	2117–2147
PPRV-RPA F2	ACTCTCAAGTACAGCGTTACYATGTTTATA	2117–2146
PPRV-RPA F3	CCAACTCTCAAGTACAGCGTTACTATGTTTATA	2114–2146
PPRV-RPA F4	ATCCAACTCTCAAGTACAGCGTTACTATGTTTATA	2112–2146
PPRV-RPA R	TCCACATCGCTGTCGTCAGATCCATCCTCTCCT	2235–2267
PPRV-RPA P	GTGAAGAGATTGAAGGACTCGAGGATGCTGAC (FAM-dT) (THF) (BHQ1-dT) CTCGTGGTTCAAGCA-C3 spacer	2156–2205

^a^PPRV-RPA F and PPRV-RPA R were defined as forward primer and reverse primer, respectively; PPRV-RPA P was *exo* probe; ^b^FAM-dT, thymidine nucleotide carrying fluorescein; THF, tetrahydrofuran spacer; BHQ1-dT, thymidine nucleotide carrying black hole quencher 1; C3 spacer to block elongation.

**Table 2:** Performance of PPRV real-time RT-RPA assay in comparison with the real-time RT-qPCR assay for detecting PPRV clinical samples (*n* = 138).

		Real-time RT-PCR results (no.)		Total	Performance characteristics (%)	
		Pos	Neg		Sensitivity	Specificity
Real-time RT-RPA results (no.)	Pos	47	2	49	100% (92.45~100%, 95%CI)	97.80% (92.29~99.73%, 95% CI)
	Neg	0	89	89		
	Total	47	91	138		

Pos, positive; Neg, negative; CI, confidence interval.

**Table 3:** Comparison of the real-time RT-RPA assay with the real-time RT-PCR assay for the detection of PPRV using clinical samples.

Sample ID	Species	Sample type	Real-time RT-PCR Threshold time (min)	Real-time RT-RPA Ct value
1	goat	nasal swab	3.00	15.14
2	goat	nasal swab	3.00	16.30
3	goat	nasal swab	7.70	30.20
4	goat	nasal swab	6.30	25.56
5	goat	nasal swab	3.00	20.16
6	goat	nasal swab	3.30	21.86
7	goat	nasal swab	3.00	21.80
8	goat	nasal swab	3.70	20.27
9	goat	nasal swab	3.70	21.75
10	goat	nasal swab	2.30	18.03
11	goat	nasal swab	3.30	19.62
12	goat	nasal swab	4.30	25.14
13	goat	nasal swab	6.00	27.27
14	goat	nasal swab	3.70	23.21
15	goat	nasal swab	2.70	19.34
16	goat	nasal swab	2.30	18.53
17	goat	nasal swab	3.00	17.82
18	goat	nasal swab	5.70	26.22
19	goat	nasal swab	4.30	18.73
20	goat	nasal swab	3.70	17.14
21	goat	nasal swab	4.00	19.55
22	goat	nasal swab	4.00	21.20
23	goat	liver	5.30	25.93
24	goat	liver	2.70	16.95
25	goat	spleen	2.70	15.27
26	goat	lung	3.00	14.47
27	goat	kidney	4.00	20.33
28	goat	lymph node	4.70	20.50
29	goat	nasal swab	5.00	23.73
30	goat	lung	3.30	15.09
31	goat	liver	4.30	22.05
32	goat	spleen	4.00	20.07
33	goat	kidney	4.30	21.46
34	goat	rectum	3.30	16.06
35	goat	lymph node	3.70	17.10
36	goat	nasal swab	16.70	Negative
37	goat	nasal swab	17.70	Negative
38	goat	nasal swab	4.70	22.74
39	goat	spleen	9.00	35.00
40	goat	lung	3.30	16.60
41	goat	lymph node	2.70	13.44
42	goat	nasal swab	2.70	15.04
43	goat	nasal swab	2.70	15.45
44	goat	nasal swab	2.70	15.30
45	goat	nasal swab	4.70	24.43
46	goat	nasal swab	4.30	22.75
47	goat	nasal swab	4.70	17.50
48	goat	liver	2.70	16.50
49	goat	lymph node	4.30	25.64

### *In vitro*-transcribed RNA

The 156-nucleotide fragment of lineage I, II and III containing the target sequence of real-time RT-RPA were synthesized and subsequently cloned into the pUC57 cloning vector (Sangon Biotech, Shanghai, China). Then, the recombinant plasmids were linearized downstream of the targeting segments. The transcript reactions were run with TranscriptAid T7 High Yield Transcription kit (Fermentas, Vilnius, Lithuania) according to the manufacturer’s instructions. The RNA transcripts made from the plasmids above containing PPRV target fragment were then treated with DNase I and purified using phenol: chloroform extraction and ethanol precipitation method according to the manufacturer’s instructions.

### Real-time RT-RPA reaction conditions

Real-time RT-RPA assay was performed in a 50 µL volume using the TwistAmp® exo RT kit (TwistDx, Cambridge, UK). The reaction mixture included 29.5 µL rehydration buffer, 3 µL extracted RNA or DNA template, 2.1 µL forward primer, 2.1 µL reverse primer, 0.6 µL probe, 0.5 µL RNase inhibitor, 9.7 µL dH_2_O and 2.5 µL magnesium acetate (280 mM). The RT-RPA reaction mixtures were incubated for 20 min at an optimized temperature (42 °C) with a brief mixing of the reaction mixtures at 230 s after the start of the incubation. The fluorescence (FAM) signal was measured every 20 s using a Twista real-time fluorometer (TwistDx, Cambridge, UK). The criteria of threshold limit for positive results were determined as described previously^24^.

### Real-time RT-PCR assay

The real-time RT-PCR assay was performed on Light Cycler 480 (Roche, Mannheim, Germany) as previously described^25^. The reactions were prepared as a 25 µL reaction volume containing 12.5 µL 2× reaction mix, 0.5 µL enzyme mix and 3 µL extracted RNA. The following thermal program was: reverse transcription at 50 °C for 15 min, followed by 95 °C for 3 min and 40 cycles of amplification (15 s at 94 °C and 1 min at 60 °C).

### Analytical sensitivity and specificity of real-time RT-RPA

To determine the detection limit of the real-time RT-RPA, serially diluted PPRV China/XJ/2013 strain viral RNAs extracted from infected cell culture supernatant with known titer ranging from 2 × 10^4^ TCID_50_/mL to 0.2 TCID_50_/mL were prepared, with RNase-free water as dilution buffer. Each dilution was assayed in eight replicates by the real-time RT-RPA. The analytical specificity of PPRV real-time RT-RPA assay was evaluated among other pathogens of goat and sheep with similar clinical signs (GTPV, *Mccp* or FMDV), other morbillivirus (MeV, CDV) and *in vitro*-transcribed RNA fragments from 4 genetically distinct lineages (lineage I, II, III and IV).

### Comparison of the real-time RT-PCR assay with real-time PCR assay using clinical samples

To explore the capability of the real-time RT-RPA assay to detect clinical specimen, the real-time RT-RPA assay for the detection of PPRV was evaluated by performance of the assay on the 138 clinical samples collected from China between 2014 to 2017 by veterinary service during outbreaks of PPR. The detection of clinical samples was carried out in triplicates. The performance of real-time RT-RPA assay was compared to that of real-time RT-PCR assay. The degree of agreement between the real-time RT-RPA and real-time RT-PCR assay results were measured with kappa value by using MedCalc software (MedCalc Software bvba, Ostend, Belgium).

### Statistical analysis

For the determination of the PPRV real-time RT-RPA assay analytical sensitivity, a semi-log regression analysis (PRISM, Graphpad Software Inc., San Diego, California) and a probit regression analysis (MedCalc Software bvba, Ostend, Belgium) were performed to calculate the detection limit of the real-time RT-RPA assay at a 95% probability level.
